# Open Minimally Invasive Parathyroidectomy Versus Minimally Invasive Video-Assisted Parathyroidectomy: A Systematic Review and Meta-Analysis

**DOI:** 10.7759/cureus.48153

**Published:** 2023-11-02

**Authors:** Mohammad Saleki, Muneer Master, Muhammad Ashhad Noor, Bako Nouri, Mohammad Alhajri, Ahmad Abul

**Affiliations:** 1 Medicine and Surgery, Queen's Hospital, London, GBR; 2 General Surgery, Royal Blackburn Teaching Hospital, Blackburn, GBR; 3 Medicine and Surgery, Manchester University National Health Service (NHS) Foundation Trust, Manchester, GBR; 4 School of Medicine, University of Leeds, Leeds, GBR

**Keywords:** thyroid and parathyroid surgery, endocrine surgery, hyperparathyroidism, minimally invasive parathyroidectomy, mivap, omip, minimally invasive surgery

## Abstract

Various minimally invasive techniques exist for surgical parathyroidectomy. The aim of this study was to conduct a meta-analysis comparing two popular minimally invasive techniques: minimally invasive video-assisted parathyroidectomy (MIVAP) and open minimally invasive parathyroidectomy (OMIP). An extensive search was conducted of online databases to identify all previous studies that had compared MIVAP and OMIP. The primary outcome measures considered were visual analog scale (VAS) score 24 hours postoperatively, conversion of operation (to open), failure rate and analgesic consumption. The data from these studies was extracted and compiled into a meta-analysis. The literature search yielded 104 studies of which four were included, enrolling 903 patients in this analysis. A significant difference was found regarding rates of conversion to open parathyroidectomy between the two groups, with the OMIP group demonstrating fewer conversions (MD = 3.52, CI = (2.04-6.08), P< 0.00001). No statistically significant differences were found between the two groups when comparing postoperative VAS scores at 24 hours (MD = -1.75, CI = (-9.8-6.3), P = 0.67), consumption of analgesia (OR = 0.49, CI = 0.07-3.54, P = 0.48) or failure rates (OR = 1.81, CI = 0.58-5.72, P = 0.31). OMIP was seen to require less need to convert to open parathyroidectomy with shorter operative times, while similar complication rates and scar lengths to MIVAP. More studies are required to evaluate the superior technique for parathyroidectomy.

## Introduction and background

The utilization of minimally invasive surgical techniques has become the predominant approach in various medical procedures. In the case of primary hyperparathyroidism, approximately 80% of cases are attributed to benign parathyroid adenomas, which lead to debilitating symptoms such as bone pain, disrupted sleep, fatigue, and anxiety [1]. The definitive treatment for this condition involves the surgical removal of the adenoma, a consideration when patients present with symptomatic manifestations or guidelines, such as serum calcium level exceeding 0.25 mmol/L above the normal range, calculated creatinine clearance below 60 mL/minute, and bone mineral density below -2.5 at the lumbar spine [2].

Historically, the preferred surgical approach for primary hyperparathyroidism was complete bilateral neck exploration (BNE), however, the introduction of minimally invasive video-assisted parathyroidectomy (MIVAP), pioneered by Miccoli [3,4], has emerged as the preferred choice among surgeons. Nonetheless, alternative minimally invasive techniques, including endoscopic, radio-guided, and open minimally invasive approaches, exist.

MIVAP swiftly gained acceptance as the standard approach to parathyroidectomy. Notably, it offered distinct advantages over BNE, primarily in terms of improved cosmetic outcomes [4,5]. Comparative studies between traditional parathyroidectomy and MIVAP have also highlighted advantages, such as significantly reduced operation time (mean 29.0+/-7.9 minutes versus 69.4+/-26.5 minutes) and lower postoperative pain scores (1.4+/-0.4 versus 2.6+/-0.6 at one hour) [6]. Consequently, surgeons have widely embraced MIVAP over BNE whenever feasible, given its theoretical reduction in complications, facilitated by enhanced visualization of adjacent structures and reduced risk (approximately 10-20%) of postoperative transient hypoparathyroidism [7].

Numerous studies and meta-analyses have consistently demonstrated the benefits of minimally invasive parathyroidectomy, including shorter operation times, enhanced cosmetic outcomes, earlier patient discharge, and reduced postoperative pain compared to traditional BNE [6,8-13]. These advantages, encompassing cost-effectiveness, reduced complication rates, diminished postoperative pain, and shorter hospital stays, are inherently tied to the minimally invasive approach itself [14]. Consequently, it is crucial to conduct comparative studies that scrutinize various minimally invasive methodologies to determine potential superiority among them.

Open minimally invasive parathyroidectomy (OMIP), introduced by Udelsmann, has gained widespread popularity for the resection of solitary parathyroid adenomas [15,16]. Although definitive conclusions regarding the superiority of MIVAP or OMIP have not been reached, a systematic review by Gracie and Hussain 2012 identified OMIP as the most favourable technique for resecting solitary adenomas causing sporadic primary hyperparathyroidism based on optimal operation times, surgeon learning curves, and cost-effectiveness, despite acknowledging that MIVAP yielded significantly better postoperative pain outcomes [17].

While numerous studies have compared these two minimally invasive techniques through both randomized and non-randomized trials, there are currently no meta-analyses available that directly compare OMIP to MIVAP. Therefore, this inaugural meta-analysis is indispensable in exploring whether one technique demonstrates superiority over the other. The findings of this study were also presented as a spotlight oral presentation at the “ASiT Annual Conference 2022: Surgical Training: The Evolution”, 4th-6th March 2022.

## Review

Methods

A systematic review was conducted following the Preferred Reporting Items for Systematic Reviews and Meta-Analyses (PRISMA) guidelines.

Eligibility criteria

Study Design

Studies must be comparative in nature, including case-control studies, randomized controlled trials (RCTs), or cohort studies and compare different surgical techniques for parathyroidectomy in patients with primary hyperparathyroidism (pHPT).

Patient Population

Patients included in the study must have a diagnosis of pHPT.

Studies should involve patients with preoperatively localized parathyroid adenoma.

Intervention

Studies must compare the two minimally invasive parathyroidectomy techniques, MIVAP and OMIP.

Outcome Measures

Studies must report on at least one of the following outcome measures: postoperative VAS score at 24 hours, conversion of operation (to open) and failure of operation, analgesic consumption, operative time, overnight stay, complications and incision/scar length.

Any studies which did not fit the inclusion criteria above were excluded from the meta-analysis.

Primary Outcomes

The primary outcomes are VAS for pain score at 24 hours postoperative, conversion of operation (to open) and failure of the procedure.

Secondary Outcomes

The secondary outcomes included analgesic consumption, operative time, overnight stay, complications and incision/scar length.

Literature Search Strategy

Two authors AA and MS independently searched the following electronic databases: MEDLINE, EMBASE, CINAHL, and the Cochrane Central Register of Controlled Trials (CENTRAL). The last search was run on 1st August 2022. Thesaurus headings, search operators and limits in each of the above databases were adapted accordingly. In addition, the World Health Organization International Clinical Trials Registry (http://appswho.int/trialsearch/), ClinicalTrials.gov (http://clinical-trials.gov/), and ISRCTN Register (http://www.isrctn.com/) were searched for details of ongoing and unpublished studies. No language restrictions were applied in our search strategies. Search terminologies included ‘’solitary adenoma’’, ‘’primary hyperparathyroidism’’, ‘’parathyroidectomy’’, ‘’minimally invasive’’, ‘’keyhole surgery’’, ‘’Video-assisted’’, ‘’open minimally invasive’’, ‘’Conventional’’. The bibliographic lists of relevant articles were also reviewed.

Study Selection

The title and abstract of articles identified from the literature searches were assessed independently by two independent authors. The full texts of relevant reports were retrieved and those articles that met the eligibility criteria of our review were selected. Any discrepancies in study selection were resolved by discussion between the authors.

Data Extraction and Management

An electronic data extraction spreadsheet was created in line with Cochrane’s data collection form for intervention reviews. The spreadsheet was pilot-tested in randomly selected articles and adjusted accordingly. Our data extraction spreadsheet included study-related data (first author, year of publication, country of origin of the corresponding author, journal in which the study was published, study design, study size, clinical condition of the study participants, type of intervention, and comparison), baseline demographics of the included populations (age and gender) and primary and secondary outcome data. Three authors (AA, MAN, MS) cooperatively collected and recorded the results and any disagreements were resolved via discussion. MM and BN then reviewed the data and synthesized the discussion.

Data Synthesis

Data synthesis was conducted using the Review Manager 5.3 software (The Cochrane Collaboration, Oxford, England). The extracted data was entered into Review Manager by two independent authors. The analysis involved was based on the fixed and random effect model. The results were reported in forest plots with 95% Confidence Intervals (CIs).

For dichotomous outcomes, the Odds Ratio (OR) was calculated between the two groups. The OR is the odds of an event in the OMIP group compared with the MIVAP group. An OR of greater than 1 would favour the OMIP group, an OR of less than 1 would favour the MIVAP group and an OR of 1 would favour neither group.

For continuous outcomes, the Mean Difference (MD) was calculated between the two groups. A positive MD would favour the OMIP group, a negative MD would favour the MIVAP group and an MD of 0 would favour neither group.

Assessment of Heterogeneity

Heterogeneity among the studies was assessed using the Cochran Q test (χ2). Inconsistency was quantified by calculating I2 and interpreted using the following guide: 0% to 25% may represent low heterogeneity, 25% to 75% may represent moderate heterogeneity, and 75% to 100% may represent high heterogeneity.

Methodological Quality and Risk of Bias Assessment

Two authors independently assessed the methodological quality as well as the risk of bias for articles matching the inclusion criteria. For randomized trials, Cochrane's tool for evaluating the risk of bias was used. Domains assessed included selection bias, performance bias, detection bias, attrition bias, reporting bias and other sources. RCT studies are classified into low, unclear and high risk of bias. For non-randomized studies, the Risk of Bias In Non-randomized Studies of Interventions (ROBINS-I) tool was used assessing seven domains with classifications including ‘’low ’’, ‘’moderate’’, ‘’serious’’, ‘’critical’’, or ‘’no information’’ if there was no information given in the article.

Results

Literature Search Results

**Figure 1:**
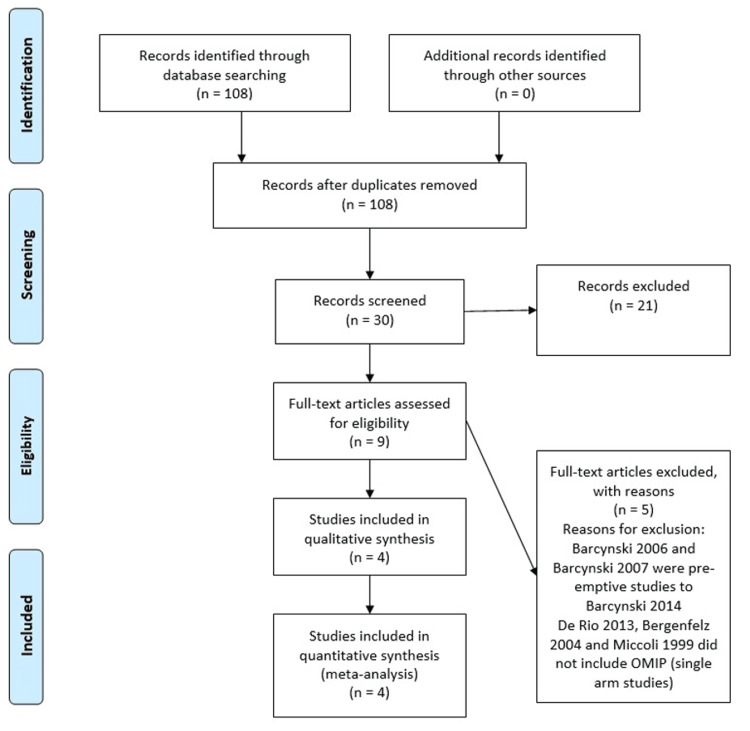
Prisma Flow diagram for selection of studies The PRISMA diagram details the search and selection processes applied during the overview. PRISMA: Preferred Reporting Items for Systematic Reviews and Meta-Analyses, OMIP: open minimally invasive parathyroidectomy

A literature search retrieved 108 articles in total which were reviewed by two independent authors to filter out duplicates, abstracts, review articles, studies without the intervention of interest as well as those without comparative control groups and reports involving non-human subjects. Four studies were selected which met the eligibility criteria, of which one was an RCT and three were observational studies (Figure 1).

Description of Studies

Garrel et al. [18] conducted a single-centre non-randomized retrospective comparative study. This study included 112 patients with pHPT who also presented with parathyroid adenoma. Patients were roughly split in half to undergo two different minimally invasive techniques of parathyroidectomy. The authors compared minimally invasive open surgery with MIVAP; 58 and 54 cases were performed respective to each technique. The comparative study of these techniques was dependent on the success and complication rates, operating time and the satisfaction of the patient and community physician.

Barczyński et al. [7] conducted a single-centre retrospective case-controlled study. A total of 455 patients with sporadic pHPT had a minimally invasive parathyroidectomy with introspective parathyroid assay performed on them. This study aimed to compare between MIVAP and OMIP, 151 and 304 patients underwent each technique respectively. Most cases (395 cases) were performed by the patient’s choice and the rest were assigned randomly. The primary outcome to measure was postoperative pain.

Hessman et al. [19] conducted a multi-centre prospective randomized trial. This study included 143 patients with pHPT. Seventy-five patients were assigned randomly to OMIP and 68 patients to MIVAP. Patients were assigned randomly in each centre using sealed envelopes in blocks of five. Patients were blinded to the randomization until the six month after the operation. The outcomes were determined by postoperative pain scoring and the duration of the operation.

In this study, Melck et al. [20] investigated the utility of MIVAP as an innovative approach for addressing pHPT in comparison to conventional OMIP, which offers similar complication rates while providing the added advantage of shorter hospital stays. A case-control study design was employed, involving patients with hyperparathyroidism exhibiting single-focus imaging results. The findings revealed that MIVAP was a viable surgical option for select patients, offering enhanced cosmetic outcomes and comparable operative times to OMIP. Moreover, it demonstrated a lower conversion rate and reduced the need for overnight hospital stays. However, patients undergoing OMIP displayed elevated preoperative calcium and parathyroid hormone levels, increased adenoma weight, and a higher incidence of long-term mortality. Despite these distinctions, the overall operative time, in-house analgesia utilization, and the occurrence of operative complications did not differ significantly between the two groups.

Risk of Bias Assessment

**Figure 2:**
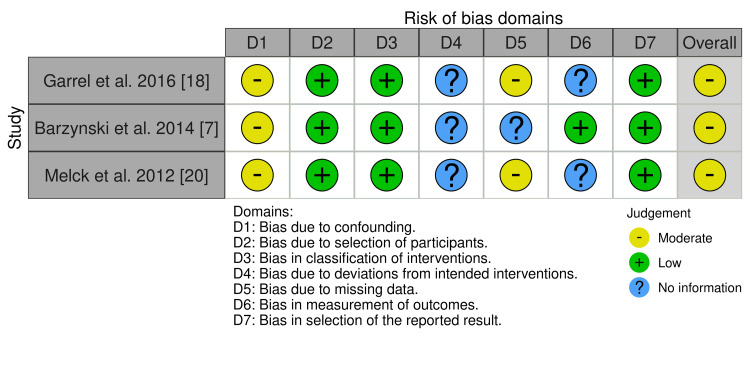
A visualization tool for ROBINS-I risk of bias assessment using the traffic light system generated by the Robvis tool. ROBINS-I: Risk of Bias In Non-randomized Studies of Interventions

We assessed the methodological quality of the Hessman et al. [19] RCT study (Table 1) using the ROB-Cochrane assessment tool, as well as the ROBINS-I for observational studies [7,18,20] (Figure 2).

**Table 1:** Assessment of Risk of Bias of the Randomized Trials using the ROB-Cochrane assessment tool for Hessman et al. 2010 [19].

First Author	Bias	Authors’ Judgement	Support for Judgement
Hessman et al. 2010 [19]	Random sequence generation (selection bias)	Low risk	Randomization was performed separately for each centre, in blocks of five using sealed envelopes
	Allocation concealment (selection bias)	Low risk	Randomization was performed separately for each centre, in blocks of five using sealed envelopes
	Blinding of participants and personnel (performance bias)	Low risk	The randomization was blinded for the patients until 6 months after the operation.
	Blinding of outcome assessment (detection bias)	Unclear risk	No information given
	Incomplete outcome data (attrition bias)	Low risk	All outcome data reported
	Selective reporting (reporting bias)	Unclear risk	No information is given regarding the study protocol
	Other bias	Low risk	Similar baseline characteristics were compared between the groups

Primary outcomes

Conversion of Operation

**Figure 3:**
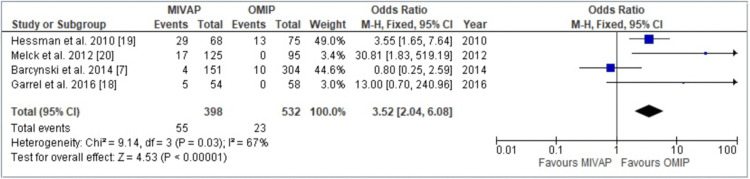
Forest plot depicting Odds Ratio (OR) between OMIP and MIVAP - conversion of operation. There was a statistically significant difference seen in the number of patients converted to open surgery [7,18-20]. OMIP: open minimally invasive parathyroidectomy, MIVAP: minimally invasive video-assisted parathyroidectomy

Figure 3 illustrates that conversion from minimally invasive procedures to other modalities (such as BNE or cervicotomy) occurred across all four studies involving a total of 930 patients. A significant difference was observed between the two groups, with a distinct preference for the OMIP group, where considerably fewer patients required conversion (OR = 3.52, 95% Confidence Interval = 2.04-6.08, p < 0.00001). Notably, there was a moderate level of heterogeneity observed among the four studies (I2 = 67%, p = 0.03).

VAS Score 24 Hours Postoperatively

**Figure 4:**
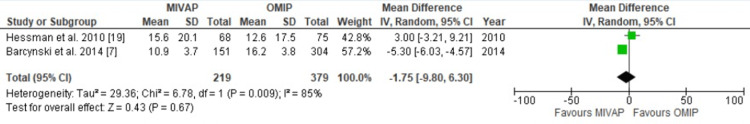
Forest plot for the mean difference between OMIP and MIVAP approaches - VAS pain score 24 hours post-operative. There was no statistically significant difference in the puncture success rate of both groups [7,19]. OMIP: open minimally invasive parathyroidectomy, MIVAP: minimally invasive video-assisted parathyroidectomy, VAS: visual analog scale

In Figure 4, VAS for pain score 24 hours after parathyroidectomy was assessed by two studies, encompassing a total of 519 patients. The standardized mean difference analysis revealed no statistically significant difference between the two groups (Mean Difference = -1.75, 95% Confidence Interval = -9.8 to 6.3, p = 0.67). A substantial level of heterogeneity among the studies was displayed (I2 = 85%, p = 0.009).

Failure Rates

**Figure 5:**
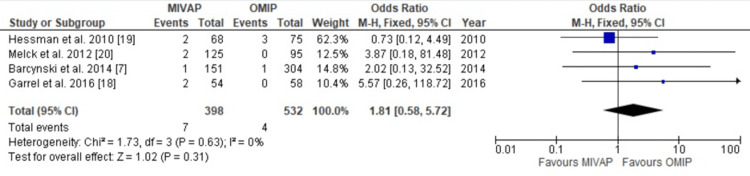
Forest plot depicting Odds Ratio (OR) analysis between MIVAP and OMIP - failure of operation. There was no statistically significant difference between the two groups in number of failed operations [7,18-20]. OMIP: open minimally invasive parathyroidectomy, MIVAP: minimally invasive video-assisted parathyroidectomy

In Figure 5, failure rates were analysed across four studies enrolling 930 patients. There was no significant difference seen between the two groups in the number of operations that failed (OR = 1.81, CI = 0.58-5.72, P = 0.31). There was low heterogeneity across the studies (I2 = 0%, P = 0.63).

Secondary outcomes

Operative Time

**Figure 6:**
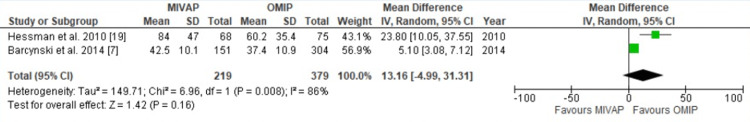
Forest plot depicting mean difference (MD) analysis between OMIP and MIVAP - operative time. There was no statistically significant difference seen in the mean difference analysis between the two groups [7,19]. OMIP: open minimally invasive parathyroidectomy, MIVAP: minimally invasive video-assisted parathyroidectomy

In Figure 6, two studies, namely Barczyński et al. [7] and Melck et al. [20], demonstrated similar operative durations in both groups (Mean Difference = 13.16, 95% Confidence Interval = -4.99-31.31, p = 0.16).

Furthermore, both Hessman et al. [19] and Garrel et al. [18] reported operative times; however, a quantitative analysis could not be performed due to the absence of mean and standard deviation data. Nevertheless, both studies indicated significant differences in operative duration, with shorter times observed in the OMIP group (p = 0.001 and p = 0.02, respectively).

Analgesic Consumption

Analgesic consumption was reported by three of the included studies including 675 patients in total. No standardized method of reporting analgesic consumption was seen between the studies hence meta-analysis was not possible. Hessman et al. [19] reported the amount of paracetamol consumed in milligrams and did not report a statistically significant difference across all timeframes (p>0.05). Melck et al. [20] evaluated the number of analgesics given and saw no significant difference between the two groups (p>0.05). Finally, Barczyński et al. [7] reported analgesic consumption at 24 hours showed a significant difference in the amount consumed between the MIVAP group consuming less mg of analgesia (89.1±48.9 vs 48.0±41.6, p<0.0001).

Length of Overnight Stay

Melck et al. [20] and Barczyński et al. [7] reported the proportion of people who stayed in hospital over a period of three days. Both studies demonstrated that MIVAP had a shorter length of hospital stay compared to OMIP. Garrel et al. [18] reported the average length of stay in days, whereby the MIVAP had a shorter mean duration of stay compared to OMIP (1.08 vs 1.37) respectively.

Complications

Garrel et al. [18] and Melck et al. [20] both documented complications within the two groups. Melck et al. [20] reported three complications in the MIVAP group, comprising one case of groin hematoma, one instance of pulmonary embolism, and one early re-operation. In contrast, the OMIP group experienced a single complication, characterized by persistent pHPT in two patients. The findings of Garrel et al. [18] for the MIVAP group included one occurrence of scalp hematoma, one case of post-operative hypercalcemia, and three instances of postoperative hypocalcaemia. On the other hand, the OMIP group exhibited two cases of postoperative hypercalcemia and two cases of postoperative hypocalcaemia [18].

Incision and Scar Length

All four studies conducted scar length comparisons between the two surgical approaches, yet meta-analysis was precluded due to variations in reporting methods. Barczyński et al. [7] and Hessman et al. [19] reported notable differences between the two groups, favouring MIVAP with significantly shorter scar lengths (p < 0.0001). Furthermore, both Melck et al. [20] and Garrel et al. [18] presented median and range data for scar length, revealing a statistically significant preference for the MIVAP group (p < 0.0001).

Discussion

In this meta-analysis comparing two minimally invasive parathyroidectomy techniques, we discovered a significantly lower conversion rate to open parathyroidectomy when OMIP was performed in contrast to MIVAP (P<0.00001). No statistically significant differences were observed in the other primary outcome measures. Regarding inter-study heterogeneity, the VAS pain score 24 hours postoperatively exhibited high heterogeneity (I2 = 85%), while the failure rate had low heterogeneity (I2 = 0%). The outcome of conversion to open surgery was determined to have a moderate level of heterogeneity (I2 = 67%), based on the assessment in the methods section.

In our analysis of secondary outcomes, we observed two notable findings. Firstly, there was a significant reduction in operative time in favour of the OMIP group, while the MIVAP group exhibited a notably smaller scar length (P < 0.00001). The shorter operative time in the OMIP group not only implies enhanced surgical efficiency but also serves to minimize the duration of anaesthesia, thereby reducing the potential for anaesthesia-related complications [21]. Conversely, the MIVAP group's smaller scar length contributes to superior cosmetic outcomes, a factor that can substantially bolster patient satisfaction [22]. These findings emphasize the importance of tailoring surgical approaches to meet individual patient needs and preferences. Striking a balance between surgical efficiency and cosmetic results becomes paramount, as it significantly influences the choice of surgical technique in managing pHPT.

Barczyński et al. [7] reported that analgesic consumption at 24 hours was lower in the MIVAP group. However, when examining the results of the other analysed studies, no significant difference was observed in this secondary outcome. This lack of substantial variance in analgesic consumption between MIVAP and OMIP techniques suggests that drawing reliable conclusions about the difference in analgesia consumption between the two approaches is challenging. Hence, further prospective studies would be required to draw conclusions about the difference in analgesia consumption.

Melck et al. [20] and Barczyński et al. [7] reported that MIVAP had a shorter length of hospital stay compared to OMIP. A shorter hospital stay was linked to better post-discharge outcomes, including reduced early readmission and mortality rates, as well as shorter readmission stays [23]. Furthermore, if a shorter hospital stay aligns with a patient's preferences, it may influence the choice of surgical technique.

At present, there is limited literature comparing the efficacy of OMIP and MIVAP and thus the overall superiority of either technique remains unclear. Both techniques are minimally invasive and have demonstrated similarly high success rates and represent excellent options for surgical parathyroidectomy, with similar efficacy and complication rates to the previous gold-standard BNE [17]. Existing studies have reported the superiority of MIVAP in terms of postoperative pain, analgesic consumption, hospital stay and cosmetic outcome [7,18,20], although the shorter duration of procedure for OMIP [19] remains an important strength. Our paper demonstrates OMIP had reduced rates of conversion surgery, and lacks superiority in only length of stay and incision of scar length as secondary outcomes, suggesting it may be a better technique than MIVAP overall. Furthermore, the cost-effectiveness of OMIP compared to MIVAP continues to be an important factor, with training in endoscopic surgery and endoscopic equipment required for the latter [24].

The use of minimally invasive techniques in surgical procedures has become increasingly popular due to the potential benefits they offer over traditional open surgery, including reduced pain, shorter hospital stays, and improved cosmetic outcomes [25]. OMIP offers better visualization of the adenoma and surrounding structures and the ability to perform BNE if necessary [7]. However at present for parathyroidectomy, MIVAP has become the most used approach [1].

The findings of this meta-analysis support the use of OMIP as the preferred minimally invasive technique for parathyroidectomy if shorter operative time and risk of conversion to open is the patient's preference. Although additional research is required to comprehensively assess the superiority of the technique, these findings provide valuable insights for parathyroid surgeons regarding the existing evidence comparing MIVAP and OMIP techniques. It is also important to note that both MIVAP and OMIP are minimally invasive techniques, and both have the potential to provide significant benefits over traditional open surgery. Therefore with this data, surgeons should continue to carefully weigh the potential benefits and risks of both techniques to make the best decision for each individual patient.

A systematic review was implemented in this meta-analysis to summarize the best available evidence regarding the efficacy of OMIP and MIVAP, and included one RCT and three retrospective studies. Initially, all studies looking at OMIP and MIVAP were included ensuring that a wide range of data was included in the meta-analysis. Independent reviewers extracted and synthesized the data ensuring a level of heterogeneity and non-biased comparison between studies. However, the reported outcomes should be viewed considering limitations. Firstly, the number of studies included in the analysis was relatively small, with only four studies enrolling a total of 903 patients. This may not be sufficient to accurately compare the two techniques, and the results of the meta-analysis should be interpreted with caution.

Secondly, it is worth noting that three of the included studies were non-randomized, introducing selection bias and potentially compromising the reliability of the results. For instance, patients in the OMIP group might have been chosen based on specific characteristics that made them better candidates for this technique, possibly leading to an overestimation of its advantages when compared to MIVAP. Furthermore, the reliance on the VAS score as a postoperative pain measurement may not have been sensitive enough to detect subtle differences between the two groups. Utilizing more precise and validated pain assessment tools like the Numeric Rating Scale or the Verbal Numeric Rating Scale could have been more appropriate in this context.

## Conclusions

In conclusion, this meta-analysis comparing OMIP and MIVAP techniques for surgical parathyroidectomy reveals several insights. OMIP demonstrates a significantly lower rate of conversion to open parathyroidectomy. While no statistically significant differences were found in other primary outcomes, secondary outcomes favour OMIP with shorter operative times, while MIVAP showed smaller scar length and length of stay. OMIP may emerge as a preferred option for parathyroidectomy, but further research is warranted to confirm its superiority and long-term efficacy. It is essential to consider the limitations of this meta-analysis, including the relatively small number of studies and the potential for selection bias in non-randomized trials. Future RCTs with greater sample sizes should strive to provide more comprehensive insights into the relative benefits and risks of these minimally invasive approaches. Ultimately, surgeons should carefully evaluate patient-specific factors to make informed decisions about the most suitable technique for individual cases.
